# Vitrectomy and external drainage of subretinal fluid containing high concentration of vascular endothelial growth factor for advanced coats disease

**DOI:** 10.1038/s41598-021-98968-9

**Published:** 2021-09-29

**Authors:** Fukutaro Mano, Itsuka Matsushita, Hiroyuki Kondo, Shoko Utamura, Chiori Kondo, Shunji Kusaka

**Affiliations:** 1 Department of Ophthalmology, Kindai University Faculty of Medicine, 377-2, Ono-higashi, Osakasayama, Osaka 589-8511 Japan; 2 Department of Ophthalmology, University of Occupational and Environmental Health, Kitakyushu, Japan

**Keywords:** Medical research, Outcomes research

## Abstract

This study investigated the surgical outcomes of Coats disease and the role of external drainage (XD) of subretinal fluid (SRF). The study is a multicenter retrospective interventional case series of 26 consecutive eyes of 26 patients who underwent surgeries for advanced Coats disease with retinal detachment. Main outcomes measured were: 1) comparison of complete SRF resolution with or without XD, and 2) variables that were associated with functional postoperative best-corrected visual acuity (BCVA) defined as BCVA of 0.1 or better, 3) intraocular vascular endothelial growth factor (VEGF) levels. Complete SRF resolution was achieved in all 14 eyes in which XD had been performed and in 75% of 12 eyes in which XD had not been performed (*P* = .03). Multivariable logistic regression analysis revealed that initial BCVA was the only variable associated with functional postoperative BCVA (odds ratio 3.24, 95% CI 0.93–11.33; *P* = .04). Markedly elevated VEGF levels were noted in the SRF compared with those in the vitreous humor (49,760 ± 52,990 vs. 707 ± 611 pg/mL, *P* = .03). XD seems to provide better anatomical success than without XD in the treatment of advanced Coats disease as XD could effectively eliminate substantial amount of VEGF in the SRF.

## Introduction

Coats disease is a non-hereditary disorder of the retinal vasculature, characterized by retinal telangiectasia, which causes subretinal and/or intraretinal exudation^1^. It usually presents unilaterally, and predominantly affects young males. Recently, Rabiolo et al. reported that subtle vascular abnormalities can be seen in the fellow eyes of Coats disease patients using ultra-widefield imaging, indicating that Coats disease is a bilateral, asymmetric condition^2^.

If not adequately treated, individuals with Coats disease may develop exudative retinal detachments, which may lead to secondary neovascular glaucoma (NVG) and eventually results in phthisis bulbi or requires enucleation to reduce pain. Drawing from clinical experience with 150 cases of Coats diseases, in 2001 Shields et al. proposed a staging system that classifies Coats disease into five categories according to severity^3^. Close observation is recommended for disease management of retinal telangiectasia without exudates (stage 1). If abnormal vessels accompany the exudative changes (stage 2), ablative therapy such as retinal photocoagulation or cryotherapy becomes the primary treatment option^4^. As intraocular vascular endothelial growth factor (VEGF) concentration is significantly increased in Coats disease^5,6^, anti-VEGF therapy alone^7,8^, intravitreal steroid implant^9^ or in combination with ablative therapy^10–12^ has been successful in reducing subretinal fluid (SRF). However, in some advanced Coats disease cases (stage 3 or higher), even multiple sessions of ablative and anti-VEGF therapy often fail to achieve SRF reduction or prevent disease progression. In these cases, surgical intervention may be required to reattach the retina^13^.

The surgical management of advanced Coats disease varies markedly, including internal or transscleral, external drainage (XD) of SRF, scleral buckling procedure, with or without vitrectomy^14^, and gas or silicone oil tamponade^15^.

We have adopted XD to avoid creating a retinal break in pediatric highly inflamed eyes. In this study, we reviewed our surgical results and investigated VEGF concentrations of aqueous, vitreous, and subretinal fluid in some eyes.

## Methods

This study was a multicenter retrospective interventional case series. The study protocol received Institutional Review Board approval from Kindai University Hospital (# 28–264, Osakasayama, Osaka, Japan) and the University of Occupational and Environmental Health (# 06–70, Kitakyushu, Fukuoka, Japan). The principles of the Declaration of Helsinki were followed. Risks, benefits, and alternative treatments were outlined with parents or guardians to obtain their consent for treatment, including the off-label use of intravitreal bevacizumab injection (IVB), and written informed consent was obtained from each parent or guardian.

Consecutive patients with Coats disease (stage 3 or higher)^3^ who underwent surgical treatment at Kindai University Hospital (Osakasayama, Japan) or Hospital of the University of Occupational and Environmental Health (Kitakyushu, Japan) between June 2009 and October 2019 with minimum postoperative follow-up of 8 months were included in this study. Patients’ history was retrospectively reviewed to obtain information concerning medical interventions (PPV, XD, ablative therapy, and IVB), visual outcomes as final best-corrected visual acuity (BCVA) and anatomical outcomes defined as complete SRF resolution, and length of the follow-up. Patients with Coats disease without surgical intervention (stage 2 or lower) or with follow-up less than 8 months were excluded. Surgeries were performed by two pediatric retina surgeons (H.K. and S.K.). Surgical approach consisted of 3-port vitrectomy with or without XD. Basically, the indication of XD was bullous retinal detachment and if there was shallow retinal detachment raising the risk of retinal tear by XD, only PPV was adopted. In general, surgery was performed after multiple sessions of ablative treatment (photocoagulation or cryotherapy) and IVB, according to the discretion of each surgeon. Postoperative ablative treatment and IVB were supplemented if an exudative retinal detachment remained with active leakage from retinal telangiectasia. Fluorescence angiography was used to monitor postoperative disease activity in patients who were cooperative to the examinations at the outpatient clinic. However, the judgment of disease activity was mainly determined through changes in SRF. The frequencies (number of sessions) of perioperative ablative therapy and IVB were also documented.

Outcomes measured were: 1) comparison of surgical results as complete SRF resolution with or without XD and their association with perioperative adjunctive treatment, and 2) variables, such as age, initial BCVA, total number of surgeries, complications, and XD that were associated with functional postoperative BCVA defined as BCVA of 0.1 or better, 3) intraocular VEGF levels with or without preoperative IVB.

### Trans-sclearl drainage and subretinal fluid collection

A sclerotomy of 2–3 mm in length was created using a Golf/Scleral Knife (MANI, Tochigi, Japan) to expose the choroid in the areas where there is sufficient elevation of the retina to drain SRF safely. Subsequently, the surgical field was dried up using surgical sponge followed by puncture of the choroid using 27-gauge needle to drain the SRF. SRF sample was collected through a 22-gauge needle-sheath catheter connected with a syringe, while being cautious to not include any blood.

### VEGF concentration analyses

Aqueous, vitreous, and SRF samples were obtained at the time of surgery from patients who underwent simultaneous PPV and XD. The samples from patients were preserved at − 80 °C and determined to analyze VEGF levels with the IRB approval. Intraocular VEGF concentrations were determined using an enzyme-linked immunosorbent assay kit (R&D Systems, Minneapolis, MN, USA) according to the manufacturer’s protocol. Minimum and maximum detectable doses of this kit are 20 pg/ml and 9,990,000 pg/ml, respectively.

### Statistical analysis

Continuous variables were summarized with their mean ± SD, and categorical variables were summarized using frequency and proportion. Surgical outcomes with or without XD were compared using the Chi-square test and Wilcoxon rank-sum test. Univariable and multivariable logistic regression analyses were used to examine the independent association between baseline parameters and functional VA. Sample size (N = 3) of VEGF analysis was determined with alpha = 0.05, beta = 0.2, and estimated average difference of VEGF level between SRF and vitreous in Coats disease as 2000 pg/ml from previous study^5,6^. The VEGF concentrations in the aqueous, vitreous, and subretinal samples were assessed using the Tukey test. Statistical analyses were performed using JMP® 15 (SAS Institute Inc., Cary, NC, USA) Version 15.1. Statistical significance was established when *P*-values were less than.05.

### Consent for publication

**Figure 1:**
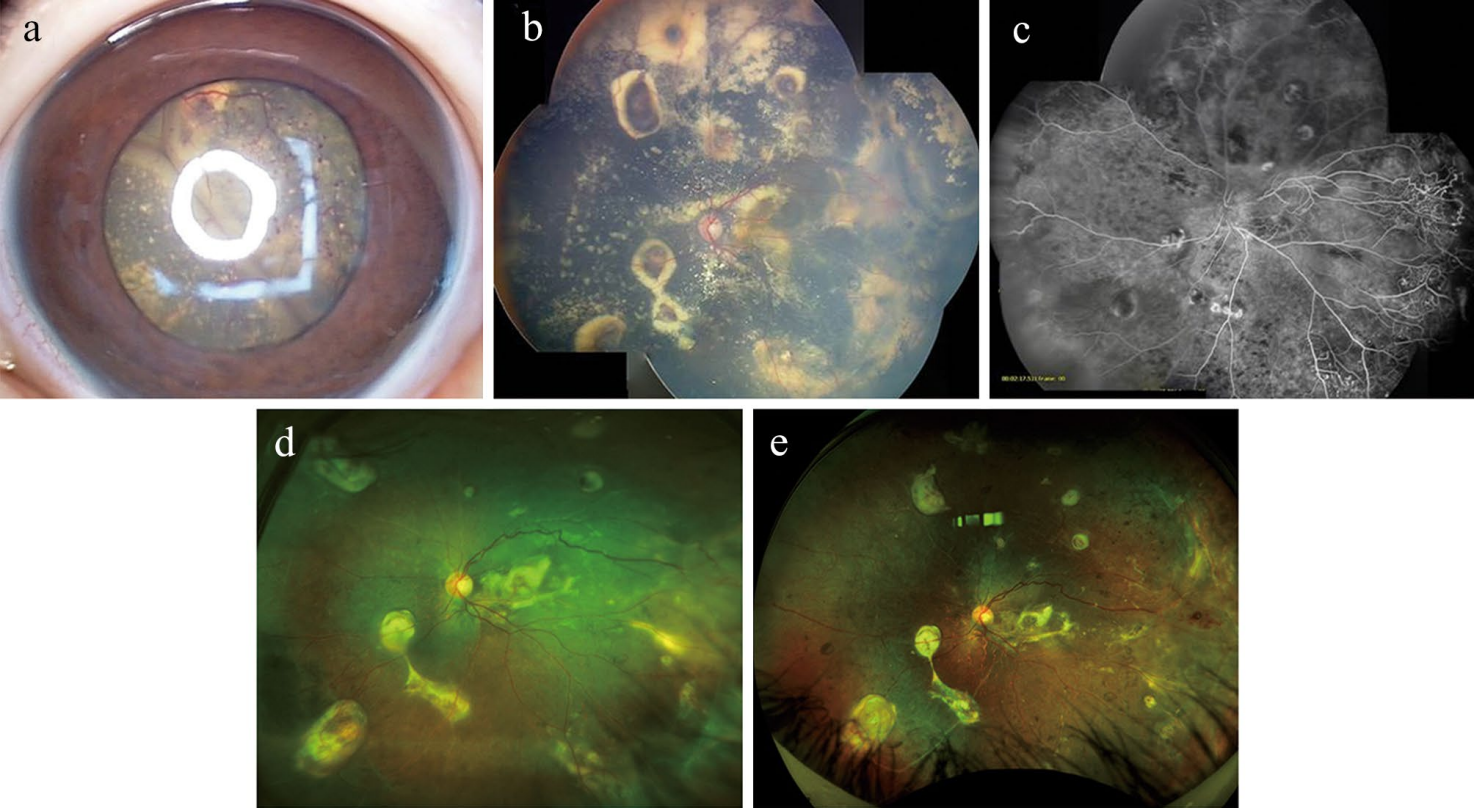
A representative case of stage 4 Coats disease. A representative case of a nine-year-old boy with stage four Coats disease. (**a**) bullous retinal detachment with telangiectatic changes can be seen behind a clear lens. (**b**) Three months after pars plana vitrectomy with external drainage, the retina was fully attached with remaining hard exudates. (**c**) Fluorescein angiography three months after the surgery shows bulb-like aneurysmal vessels, which were ablated by photocoagulation. (**d**) Wide-field fundus photograph three years after the surgery shows the attached retina with regressed hard exudates. (**e**) Fundus photo seven years after the surgery shows complete resolution of lipid exudates with subretinal fibrosis around the aneurysms.

The authors affirmed that the legal representatives provided informed consent for publication of the images in Fig. 1a–e.

## Results

### Baseline characteristics

Twenty-six eyes of 26 patients with Coats disease were enrolled. In the presenting cases, 25 patients were male and one patient was female. The mean age was 9.1 ± 5.4 (range 0.6–19) years old at the time of surgery. There were five cases with stage 3A, 18 cases with stage 3B, and three cases with stage 4 Coats disease. The overall mean follow-up period was 41.4 ± 32.9 months (range 8–126 months).

### Visual and anatomical outcomes and complications

Overall, baseline logMAR VA (1.8 ± 1.0, range 0.1–2.9) significantly improved at the final follow-up (1.6 ± 1.0, range 0.22–2.9; *P* = 0.02). All patients received PPV with (14 patients) or without (12 patients) XD. The treatment course along with the baseline characteristics are summarized in Table 1. During surgery, one patient experienced an iatrogenic retinal break during peripheral vitreous shaving (case 20). Proliferative vitreoretinopathy (PVR) (case 9), cataract formation (case 16), corneal opacification (case 23), and two cases of pupillary closure (case 21 and 25) were observed postoperatively. One case (case 22) had transient ocular hypertension related to silicone oil tamponade, which was successfully controlled by topical glaucoma eyedrops. No cases required enucleation. There was no evidence of systemic complications regarding the use of IVB.

**Table 1:** Baseline characteristics, treatment course, and the functional and anatomical outcomes.

Case	Sex	Age	Stage	Total number of surgeries	Intra-op procedures	Tamponade	XD	Initial BCVA	Final BCVA	Pre-op IVB	Post-op IVB	Pre-op ablation therapy	Post-op ablation therapy	Complete SRF resolution	Complications	Follow up (months)
1	M	10	3B	1	V, PC	SF_6_	No	0.8	0.6	1	0	2	0	Yes	None	78
2	M	3	4	1	V, PC, IVB	None	Yes	NLP	LP	0	0	0	1	Yes	None	62
3	M	11	3A	2	V, PC	Air	No	0.9	0.6	0	0	5	0	Yes	None	46
4	M	10	3B	2	V, PC	Air	Yes	LP	NLP	1	0	0	0	Yes	None	38
5	M	1	3B	1	V, PC	None	Yes	NLP	LP	0	0	0	2	Yes	None	39
6	M	8	3B	1	V, PC	None	No	0.15	0.2	1	1	1	0	Yes	None	46
7	M	0.6	3B	2	V, PC, IVB	None	Yes	NLP	HM	0	1	0	2	Yes	None	36
8	M	6	3A	1	V, PC, IVB	None	No	0.03	0.03	0	1	1	1	Yes	None	35
9	M	1	3A	3	V, PC	None	No	LP	LP	2	2	2	4	No	PVR	48
10	F	14	4	6	V, PC	SO	Yes	LP	0.09	1	0	0	5	Yes	None	24
11	M	15	3B	1	V, PC	None	Yes	0.03	0.15	1	0	1	0	Yes	None	20
12	M	9	3B	1	V, PC	None	Yes	LP	HM	4	0	5	0	Yes	None	19
13	M	12	3A	1	V, PC, IVB	None	No	0.2	0.6	5	3	2	1	Yes	None	13
14	M	19	3B	3	V, PC	SF_6_	Yes	0.15	0.3	0	1	0	2	Yes	None	8
15	M	9	3B	4	V, PC, IVB	None	Yes	0.03	0.05	0	0	2	3	Yes	None	26
16	M	8	3B	1	V, PC	None	Yes	HM	0.1	0	0	0	0	Yes	cataract*	22
17	M	12	3B	1	V, PC, IVB	None	No	0.1	0.4	2	1	6	1	Yes	None	22
18	M	11	3B	3	V, PC, IVB	None	No	0.15	0.03	2	3	7	3	No	None	24
19	M	9	3A	1	V, PC, IVB	None	No	0.07	0.07	1	0	6	0	Yes	None	18
20	M	1	4	2	V, PC, Cryo	SO	Yes	Unknown	Unknown	0	0	3	0	Yes	Iatrogenic retinal break	9
21	M	2	3B	4	V, PC, IVB	SO	Yes	Unknown	NLP	0	0	0	0	Yes	Pupil closure	117
22	M	11	3B	4	V, PC, IVB	SO	Yes	HM	NLP	0	1	0	0	Yes	Glaucoma	108
23	M	15	3B	5	V, PC	None	Yes	Unknown	LP	0	0	0	0	Yes	Corneal opacity	126
24	M	19	3B	2	V, PC	None	No	HM	HM	1	0	1	0	Yes	None	10
25	M	6	3B	4	V, PC	C_3_F_8_	No	NLP	HM	0	0	0	0	No	Pupil closure	60
26	M	14	3B	1	V, PC	None	No	HM	0.4	0	0	0	0	Yes	None	22

M; male; F; female, SRF; subretinal fluid, IVB; intravitreal bevacizumab injection, V, vitrectomy, PC; laser photocoagulation, SF_6_; sulfur hexafluoride gas, C_3_F_8_; perfluoropropane gas, SO; silicone oil, op; operative, XD; external subretinal fluid drainage, BCVA; best-corrected visual acuity, HM; hand motion, LP; light perception, NLP; no light perception, PVR; proliferative vitreoretinopathy.

*Cataract developed during the surgery as trocar insertion caused the lens damage.

### Surgical outcomes with or without external subretinal fluid drainage

Comparison analyses of patients who received PPV with XD (XD group) and PPV without XD (No XD group) are summarized in Table 2. Because XD was used mainly in eyes with bullous exudative retinal detachments, baseline BCVA was significantly worse in the XD group than No XD group (2.3 ± 0.7 vs. 1.4 ± 1.0; *P* = 0.01). Subsequently, the mean final BCVA of XD group was worse than that of No XD group (2.0 ± 0.9 vs. 1.1 ± 0.9; *P* = 0.02). Nevertheless, the number of postoperative IVB injections tended to be less in the XD group than that in the No XD group (0.2 ± 0.4 vs. 0.9 ± 1.2), although this was not statistically significant (*P* = 0.08). In addition, complete SRF resolution was achieved in all cases in the XD group (Fig. 1), while only 75% of cases achieved complete resolution in the No XD group (*P* = 0.03). More complications were observed in the XD group, including cataract formation, an iatrogenic retinal break during peripheral vitreous shaving, corneal opacification, pupillary closure, and transient ocular hypertension. However, there was no evidence that these complications were related to the XD procedure.

**Table 2:** Comparison of surgical outcomes for vitrectomy with or without external subretinal fluid drainage.

	External drainage Yes (N = 14)	External drainage No (N = 12)	*P* value
Age	8.4 ± 6.1	9.9 ± 4.5	0.48
Sex (Male:Female)	13:1	12:0	0.26
Stage (3A:3B:4)	0:11:3	5:7:0	**0.01**
Initial VA (logMAR)	2.3 ± 0.7	1.4 ± 1.0	**0.01**
Final VA (logMAR)	2.0 ± 0.9	1.1 ± 0.9	**0.02**
Post-op ablation therapy	1.1 ± 1.5	0.8 ± 1.3	0.80
Post-op IVB	0.2 ± 0.4	0.9 ± 1.2	0.08
Total number of surgeries	2.6 ± 1.7	1.8 ± 1.1	0.16
Complete SRF resolution	14/14 (100%)	9/12 (75%)	**0.03**
Complications	5/14 (36%)	2/12 (17%)	0.27
Follow-up period (Month)	46.7 ± 40.6	35.2 ± 9.5	0.38

IVB, intravitreal injection of bevacizumab; SRF, subretinal fluid.

Significant P values in bold.

### Variables associated with functional VA

Postoperative functional VA defined as 0.1 or better was associated with older age at surgery (odds ratio [OR] 0.79 per 1 SD, 95% CI 0.63–0.99; *P* = 0.02) and better initial BCVA (OR 3.85 per 1 SD, 95% CI 1.24–11.93; *P* = 0.008) (Table 3). Multivariable logistic regression analysis incorporating the strongest univariate analysis variables revealed that better baseline BCVA was the only parameter associated with postoperative functional VA (OR 3.24 per 1 SD, 95% CI 0.93–11.33; *P* = 0.04).

**Table 3:** Variables associated with functional visual acuity.

	OR	95% CI	P-value
**Univariable logistic regression analysis**			
Age	0.79	0.63–0.99	**0.02**
Initial BCVA	3.85	1.24–11.93	**0.008**
Total number of surgeries	1.46	0.78–2.72	0.21
Complications	4.5	0.44–46.17	0.21
XD	2.25	0.44–11.52	0.33
**Multivariable logistic regression analysis**			
Age	0.78	0.58–1.06	0.06
Initial BCVA	3.24	0.93–11.33	**0.04**

OR, odds ratio; CI, confident interval; P, probability; BCVA, best-corrected visual acuity; XD, external drainage of subretinal fluid.

Significant P values are shown in bold.

### VEGF levels in aqueous, vitreous, and subretinal fluid

Aqueous humor, vitreous humor, and SRF samples were obtained at the time of surgery from seven patients who underwent PPV and XD simultaneously. Subretinal samples were carefully collected without blood contamination. VEGF levels of all samples were within the detectable range. Two patients received IVB within two months prior to the PPV. VEGF levels in aqueous humor, vitreous humor, and SRF were measured and compared (Table 4). Markedly high VEGF levels were observed in SRF compared with those of the vitreous humor (49,760 ± 52,990 vs. 707 ± 611 pg/mL, *P* = 0.03), whereas no difference was observed between VEGF levels in the aqueous humor and those in the vitreous humor (1,060 ± 676 vs. 707 ± 611 pg/mL, *P* = 0.5). Notably, the two cases (case 22 and 24) that received preoperative IVB (1 month and 2 months before surgery, respectively) still exhibited high VEGF levels (1,420 and 4,900 pg/mL) in the SRF after surgery.

**Table 4:** Intraocular VEGF levels associated with intravitreal injection of bevacizumab.

Case number	Gender	Age	Stage	Pre-op IVB (time of injection)	VEGF level (pg/mL)		
					Aqueous humor	Vitreous humor	Subretinal fluid
12	M	9	3B	Yes (5 months before)	N/A	49	112,000
15	M	9	3B	No	N/A	581	23,700
16	M	8	3B	No	N/A	185	3,119
20	M	2	3A	No	N/A	597	75,820
22 (1st)	M	11	3B	No	1775	1,160	39,100
22 (2nd)	M	11	3B	Yes (1 month before)	370	N/A	1,420
23	M	15	3B	No	430	N/A	138,000
24	M	19	3B	Yes (2 months before)	970	1,670	4,900

op; operative; IVB, intravitreal injection of bevacizumab; VEGF, vascular endothelial growth factor; M male; m, month; N/A, not available.

## Discussion

The aim of this study was to retrospectively investigate the role of XD in surgical outcomes of advanced Coats disease. Complete SRF resolution was achieved in all cases in which SRF was drained externally, whereas only 75% of cases achieved complete SRF resolution with PPV alone. We also noted considerably higher VEGF levels in the SRF compared with those of aqueous and vitreous humors, even after preoperative IVB.

In 1988, Silodor et al.^16^ reported about the successful treatment of seven children with advanced unilateral Coats disease with a total bullous exudative retinal detachment who had undergone XD and cryotherapy and compared them with six untreated children, four of whom developed NVG. None of these seven eyes treated has developed painful NVG, and the retina was fully attached in three eyes and partly or totally detached but with shallow SRF in three eyes. One child was lost to follow-up. They concluded that XD could halt disease progression and prevent the development of NVG^16^. Recently, Li et al. reported that ablative therapy in combination with PPV and XD was effective in preventing disease progression in 16 patients with stage 3B Coats disease with 5 years follow-up^17^. At final follow-up, 75% (12/16) of the stage 3B Coats disease cases in this series demonstrated no progression to stage 4 or stage 5. However, 4 cases developed NVG or phthisis bulbi or both.

Our results showed that even though more severe cases were enrolled in the XD group, complete SRF resolution was achieved in all cases. However, in the No XD group, complete SRF resolution was achieved in only 75% of cases. Moreover, the number of required postoperative IVB sessions tended to be less in the XD group. Taken together, XD appears to provide better surgical outcomes possibly by eliminating highly concentrated SRF’s VEGF, which is likely to be reduced poorly by IVB.

It is controversial whether PPV should be performed in combination with XD. Stanga et al.^18^ proposed that the use of fine needle trans-scleral drainage of SRF with simultaneous anterior chamber infusion is less invasive than vitrectomy. In their study including eight cases of Coats disease with a mean follow-up of 32.9 months, they reported complete resolution of SRF with no perioperative complications^18^. The same group also reported that a multiple therapy approach consisting of surgical drainage of SRF with simultaneous IVB and laser photocoagulation sessions achieved complete SRF reabsorption in two patients with stage 3 Coats disease^19^. Muftuoglu et al. reported successful vitrectomy followed by internal drainage of SRF and long-term silicone oil tamponade in 5 Coats disease patients^20^. According to the authors, this approach also halted disease progression and avoided painful NVG development. However, there is a concern that internal drainage may be associated with postoperative PVR development. In a literature review by Sigler et al.^13^, it is suggested that internal drainage should be avoided because retinal breaks may lead to a persistent total exudative or rhegmatogenous retinal detachment as a result of underlying exudates and RPE deficiency in eyes with Coats disease. Cai et al. evaluated the efficacy of endolaser photocoagulation by a two-port pars plana nonvitrectomy approach^14^. Twenty-four out of the 25 treated eyes (96%) had retinal reattachment, and telangiectasias of the 24 eyes was resolved with no severe complications. In our study, XD was performed in combination with PPV, and internal drainage was never performed. A single case of an iatrogenic retinal break was noted as a complication in our study, which was successfully treated. Due to the low rate of complications during surgery, we believe that PPV with XD is relatively safe, and could provide the advantage of clearing various cytokines from the vitreous^21^. Also, there may be a benefit in performing simultaneous direct photocoagulation to abnormal vessels immediately after XD.

We also investigated the predictive factors associated with functional final VA. In short, multivariate logistic regression analysis revealed that the initial BCVA was the only variable independently associated with functional VA. Parks et al.^22^ evaluated the efficacy of IVB combined with laser photocoagulation in the treatment of adult-onset Coats disease. Among 13 patients with a mean follow-up period of 24.8 months, final BCVA was significantly correlated with baseline BCVA (*P* < 0.001; r = 0.882). Although their patients’ population differed from ours, particularly with respect to ages and races, their findings were similar in that the initial VA was likely to be associated with the final VA. Furthermore, in our study, older age tended to be associated with good final VA, although the association was not statistically significance (*P* = 0.06). In other words, surgery for Coats disease of younger-aged patients may be associated with worse prognosis. Similarly, Daruich et al.^23^ reviewed data for 98 patients with Coats disease and found that younger age at diagnosis was correlated with more severe disease stage (*P* < 0.0001, r =  − 0.52) and worse visual outcome. Dalvin et al. evaluated 351 patients with Coats disease, classifying them into three age categories: ≥ 3 years, 3 ≤ 10 years, and > 10 years. They found that at the end of follow-up (mean 70 months), the youngest patient group had poorer VA outcome (< 20/200: 83% vs. 64% vs. 39%; *P* < 0.001), less disease resolution (43% vs. 65% vs. 62%; *P* = 0.01), and were more likely to ultimately require enucleation (22% vs. 10% vs. 6%; *P* = 0.01)^24^. Therefore, age should also be considered a prognostic marker in Coats disease.

He et al.^6^ reported considerably elevated subretinal VEGF levels in four eyes with Coats disease (mean 2,394 pg/mL) compared with those of five eyes with rhegmatogenous retinal detachment (mean 15 pg/ mL). They also noted a reduction in aqueous VEGF from 1,247 to 20 pg/mL in one eye after treatment with IVB. Similarly, Zhao et al.^25^ and Feng et al.^26^ observed that aqueous VEGF concentrations correlated with the severity of Coats disease. As shown in these previous studies, the location of elevated VEGF is skewed in Coats disease; specifically, SRF contains much more abundant VEGF. However, the exact reason why the subretinal VEGF is higher than the vitreous or aqueous humor is unclear and to our knowledge no study has revealed the subretinal dynamics of VEGF levels associated with anti-VEGF administration.

In the current study, markedly elevated VEGF levels were noted in the SRF compared to the vitreous humor in advanced Coats disease. Interestingly, two patients that received IVB within 2 months before surgery still showed high VEGF levels in their SRF. There are several possible reasons why their subretinal VEGF levels were high despite preoperative IVB: 1) there were substantially high levels of VEGF in the subretinal space that could not be suppressed by IVB, 2) anti-VEGF agents, especially bevacizumab that has a relatively high molecular weight (MW) may infiltrate poorly into the subretinal space. Mordenti et al.^27^ examined the intraocular tissue distribution and pharmacokinetics in Rhesus monkeys following intravitreal injection of ^125^I-labeled full-length antibody (MW: 148 kDa) and Fab antibody fragment (MW: 48.3 kDa). Microradiography demonstrated that the full-length antibody did not penetrate the internal limiting membrane of the retina. In contrast, the Fab antibody fragment diffused through the neural retina to the retinal pigment epithelium^27^.

Given that substances with smaller MW penetrate the retina more effectively, ranibizumab (MW: 48 kDa), aflibercept (MW: 115 kDa), and brolucizumab (MW: 26 kDa) might be more effective than bevacizumab (MW: 149 kDa) for the treatment of Coats disease. However, to the best of our knowledge, no experimental or clinical evidence is available regarding this matter.

A previous histopathological study using the enucleated eyes of end-stage Coats disease patients showed that cholesterol crystals and lipid-laden macrophages exist in the subretinal space^28^. Kase et al. evaluated VEGF immune-reactivity in enucleated eyes and found a considerably high amount of VEGF-positive macrophages in the subretinal space and detached retina with retinal vessel abnormalities. The study indicated that abnormal vessels trigger VEGF production through increased immunoreaction of the VEGF receptor^29^. Besides, peripheral capillary dropout was observed in the patient with Coats disease, which also increases VEGF^30^. Su et al. examined the VEGF concentration of SRF collected from 31 eyes with rhegmatogenous retinal detachment and compared it with that in 8 eyes with PVR. The average VEGF level in SRF was 355 pg/mL in eyes with simple rhegmatogenous retinal detachment and 901 pg/mL in eyes with PVR. The authors suggested that the relative retinal ischemia in the detached retina increases VEGF release into the subretinal space^31^. In the present study, the mean VEGF concentration of subretinal fluid collected from patients with advanced Coats disease was 49,760 pg/mL, which was 55 times higher than the amount of subretinal fluid collected from PVR patients. The viscous protein rich SRF possibly interrupts the transport of oxygen and nutrients between the retina and retinal pigment epithelium and choroid. The detached retina then becomes ischemic, accelerating further production of VEGF. Therefore, in combination with anti-VEGF therapy and PPV, XD may halt the vicious cycle of VEGF production and facilitate the efficacy of ablative therapy. Limitations of this study include a relatively small number of cases and the retrospective nature of the surgical outcome analyses. Baseline characteristics of disease severity differed between XD and No XD group, i.e., the XD group enrolled patients with stages worse than the No XD group. In addition, the decision of performing XD was at the surgeon’s discretion based on the disease conditions and not randomized. Sample size for VEGF analysis was not initially determined. Furthermore, VEGF level analyses were performed in a select number of patients who underwent XD as it is technically difficult to collect subretinal samples from patients without XD.

In conclusion, XD with PPV provided better anatomical success than vitrectomy without XD. Despite the preoperative intravitreal injection of anti-VEGF agent, abundant VEGF remained in the subretinal space. As external drainage may eliminate VEGF-rich SRF, external drainage with PPV is recommended to achieve complete resolution of SRF and prevent disease progression.

## Data Availability

The authors confirm that the data supporting the findings of this study are available within the article.
